# Family Well‐Being During the COVID‐19 Pandemic: The Risks of Financial Insecurity and Coping

**DOI:** 10.1111/jora.12776

**Published:** 2022-06-24

**Authors:** Marybel R. Gonzalez, Sandra A. Brown, William E. Pelham, Stefanie C. Bodison, Connor McCabe, Fiona C. Baker, Arielle Baskin‐Sommers, Anthony Steven Dick, Gayathri J. Dowling, Sabrina Gebreselassie, Mathieu Guillaume, Andrew T. Marshall, Chandni Sheth, Elizabeth R. Sowell, Amandine Van Rinsveld, Susan F. Tapert

**Affiliations:** ^1^ University of California; ^2^ University of Florida; ^3^ University of Washington; ^4^ SRI International; ^5^ Yale University; ^6^ Florida International University; ^7^ National Institute on Drug Abuse; ^8^ Northwestern University; ^9^ Stanford University; ^10^ Children's Hospital Los Angeles; ^11^ University of Southern California; ^12^ University of Utah

**Keywords:** financial insecurity, family well‐being, coping, COVID‐19 pandemic

## Abstract

During the COVID‐19 pandemic, families have experienced unprecedented financial and social disruptions. We studied the impact of preexisting psychosocial factors and pandemic‐related financial and social disruptions in relation to family well‐being among *N* = 4091 adolescents and parents during early summer 2020, participating in the Adolescent Brain Cognitive Development^SM^ Study. Poorer family well‐being was linked to prepandemic psychosocial and financial adversity and was associated with pandemic‐related material hardship and social disruptions to routines. Parental alcohol use increased risk for worsening of family relationships, while a greater endorsement of coping strategies was mainly associated with overall better family well‐being. Financial and mental health support may be critical for family well‐being during and after a widespread crisis, such as the COVID‐19 pandemic.

## INTRODUCTION

The COVID‐19 pandemic brought on unprecedented and severe financial insecurity and disruption to social routines for many families across the United States (Bell & Blanchflower, 2020; Prime et al., 2020; Sohrabi et al., 2020). In summer 2020, 26% of households expected some loss of income; 10% of families endorsed food insecurity; and 7% housing insecurity, as unemployment rates peaked at 15% (U.S. Census Bureau, 2020). COVID infection mitigation strategies such as social distancing, school closures, and group activity restrictions disconnected people from social support and routines that typically maintain well‐being (Sun et al., 2020; Volk et al., 2021). The widespread nature of the crises resulted in a naturalistic social experiment (Lebow, 2020), with some families adopting healthy coping strategies to relieve stress and withstanding potential pandemic‐related adversity, and others struggling to survive pandemic‐related challenges (Veldhuis et al., 2021; Walsh, 2016).

The Family Adjustment and Adaptation Response (FAAR) model posits that family well‐being, that is the adaptation of relationships and functioning between family members in response to challenges, is a crucial component for how youth are supported during and after exposure to adversity (Patterson, 1988, 1993, 2002; Walsh, 2016). The FAAR model emphasizes that when faced with challenges, the family as a unit engages in adaptive processes that strive to bring equilibrium to family relationships and that these processes involve balancing the demands (i.e., changes and disruptions of the COVID‐19 pandemic) with family capabilities (i.e., psychosocial health and coping strategies). Notably, studies have demonstrated that the experience of struggle for families can help them emerge stronger, with better resources for adapting to future challenges (Walsh, 2016). Therefore, it is important to understand the risk and promotive factors for healthy family well‐being during the pandemic, including how major changes and disruptions (i.e., financial insecurity and social disruptions) may have challenged families, and the influence of coping strategies (Masten & Motti‐Stefanidi, 2020).

Since the start of the pandemic, many families have experienced compounded burdens, including financial and social distress due to restrictions, lockdowns, and changes in routines (Kantamneni, 2020; Park et al., 2020). While social connections can mitigate stress (Zaki & Craig Williams, 2013), social distancing and restrictions led to limited access to family and friends, potentially adding to the burden on parents. Increases in caregiving burden have been related to more severe parental stress and poorer mental health, greater parent–child conflict, and less closeness (Cluver et al., 2020; Masten & Narayan, 2012; Park et al., 2020; Russell et al., 2020). Such findings are consistent with the FAAR model, signaling shifting of dynamics of family relationships during the pandemic as the family unit attempts to balance resources, including coping strategies, with demands (Patterson, 2002).

Parents play a critical role in promoting well‐being for their family and their health and ability to cope with stressors may have a direct effect on family well‐being (Cox & Paley, 1997; Newland, 2015; Park et al., 2020; Russell et al., 2020). Coping involves cognitive and behavioral responses to adjust to the demands of internal and external stressors (Folkman & Moskowitz, 2012). During the pandemic, adults have endorsed active coping activities (i.e., taking steps to reduce stress and seeking socio‐emotional support), suggesting adaptive responses to stress (Aldwin, 2007; Baumeister & Leary, 1995; Park et al., 2020). In particular, healthy active coping strategies such as exercise and making time to relax have been associated with better mood and less psychosocial distress during the pandemic (Bateman et al., 2021; Brand et al., 2020; Conversano et al., 2020; Rodríguez‐Rey et al., 2020).

In contrast, maladaptive or avoidance coping strategies (e.g., use of alcohol) reflect an attempt to escape the stressor and may have an adverse consequence for family well‐being (Aldwin, 2007). Alcohol use among adults increased during the pandemic (Czeisler et al., 2020; Rodriguez et al., 2020), but its relation to family well‐being has been less studied (Schmits & Glowacz, 2021; Wardell et al., 2020). Those who experienced greater demand on resources during the pandemic are at higher risk for maladaptive coping (Veldhuis et al., 2021; Vetter et al., 2008; Vinkers et al., 2020; Wardell et al., 2020). While COVID mitigation recommendations have been clear, relatively less public health focus has centered on strategies for families to effectively cope with stress and social disruptions (Otu et al., 2020; Sameer et al., 2020; World Health Organization, 2020).

Adults with a history of anxiety and depression have been reported to be more susceptible to pandemic‐related stress, a potential risk for family strife (Veldhuis et al., 2021; Vinkers et al., 2020). Histories of parental substance abuse, exposure to family violence, or material hardship have predicted increased family violence during disasters (Seddighi et al., 2021). As in previous times of crisis, financial insecurity during the pandemic (e.g., wage and job loss and food or housing insecurity) induced stress on parents and families, decreasing resources critical for family well‐being (Gassman‐Pines et al., 2020; Prime et al., 2020; Williams & Cheadle, 2016). Previous studies demonstrated that during disasters, families most vulnerable to food and housing insecurity were at the greatest risk for domestic violence (Seddighi et al., 2021). However, according to the FAAR model, even families with potential vulnerabilities, such as those with prepandemic financial and psychosocial hardships, can adapt strategies to cope, grow, and emerge stronger (Walsh, 2015). Thus, it is important to investigate the potential influence of prepandemic financial and psychosocial hardships on family well‐being during the COVID‐19 pandemic.

For parents of adolescents, in particular, the COVID‐19 pandemic presented unique challenges given the socio‐emotional developmental changes that youth were undergoing, with transitions toward independence and personal development (Steinberg & Silk, 2002). We studied a large sample of demographically diverse families, specifically parents of adolescents, to investigate the influence of pandemic‐related financial and psychosocial disruptions on family well‐being (i.e., parent perception of stress due to the pandemic, ability to enjoy life, family stress, and conflict). We tested how family well‐being during the pandemic was predicted by (1) prepandemic economic and psychosocial vulnerability, (2) pandemic‐related financial insecurity, (3) pandemic‐related disruptions to social support and routines, and (4) coping activities during the pandemic. First, we hypothesized prepandemic history of financial insecurity and poorer psychosocial well‐being among parents would be associated with poorer family well‐being during the pandemic. Second, we hypothesized that severity of financial insecurity (independent of any prepandemic financial or psychosocial hardship) and disrupted social support and routines would be associated with poorer family well‐being. Third, we hypothesized coping strategies would be associated with more positive family well‐being while parental alcohol use coping would be associated with poor family well‐being. When faced with adversity, how a family responds is important for youth outcomes, with youth faring the best in the aftermath of a crisis with better family well‐being (Masten & Motti‐Stefanidi, 2020; Walsh, 2016). Thus, investigating how family well‐being is affected by prepandemic psychosocial factors and pandemic‐related financial and social disruptions, as well as coping strategies, is important for understanding the family factors that may positively support youth during and after a crises such as the COVID‐19 pandemic (Newland, 2015; Tramonti, 2021; Verger et al., 2021).

## METHOD

### Participants

We studied 4091 youth and parents (i.e., biological or legal guardians) who participated in the Adolescent Brain Cognitive Development ^SM^ (ABCD) Study COVID‐19 Survey. ABCD is a study of 11,878 youth enrolled at 9–10 years of age, followed at 21 sites across the United States. Data reported here were obtained from the ABCD COVID‐19 Survey First Data Release (DOI: 10.15154/1520584). Families in which the parent/legal guardian provided written consent and permission and the youth provided assent to participate in the ABCD study were invited via email or text to complete an ABCD COVID‐19 Survey. The COVID survey data were linked to prepandemic ABCD study data from the most recent available timepoint collected between September 2018 and January 2020 from either the baseline, 1‐year follow‐up, or 2‐year follow‐up visit (data available in the ABCD data release 3.0 DOI: 10.15154/1519007; Barch et al., 2018).

### Study Design

Three COVID‐19 survey waves were sent out to parents and youth (May 2020, June 2020, and August 2020) and were compensated $5 each, with most participants completing the survey within the first 2 weeks of sending. We analyzed COVID data from the June 2020 survey for which family well‐being questions were administered to parents and youth. A total of 5254 youth completed the COVID survey, of which 4091 youth had available data for both the COVID parent survey and prepandemic measures (see Appendix S1: Figure S1 for more details).

### Measures

#### Pandemic family well‐being principal components (PCs)

Seven items from the COVID survey were used to index family well‐being, including COVID‐related youth report of (1) *quality of family relationships* (Likert scale 1 to 5); (2) *frequency of participation in family activities* (Likert scale 0 to 4); (3) *frequency of communication with parents* (Likert scale 1 to 5); (4) *frequency of communication with siblings* (Likert scale 1 to 5); (5) *tone of communication with parents* (Likert scale 1 to 5) with higher scores indicating more positive change; (6) *tone of communication with siblings* (Likert scale 1 to 5) with higher scores indicating more positive change; and (7) *parent report of family stress and discord* (Likert scale ranging from 0 to 3) with higher scores indicating more stress and discord. More details about the questions and response options are provided in Appendix S1 Table S1. Summary measures of family well‐being were then derived by applying a principal component analysis (PCA) with all 7 items using pcaMethods, an R‐package that conducts PCAs with imputation for missing data. The svdImpute algorithm was used to estimate missing values (Stacklies & Redestig, 2007; Troyanskaya et al., 2001) by finding an optimal linear combination by regressing the incomplete variables against the k most significant loadings. The svdImpute can tolerate a large percentage of missing data (> 10%).

#### Prepandemic measures of psychosocial vulnerability

##### Material hardship

During a prepandemic visit, parents completed a 5‐item questionnaire to assess material hardship, including questions on whether families were unable to afford food, had experienced eviction or lapse in rent/mortgage, or were unable to afford a phone or utility services were turned off due to lapse in payment (Barch et al., 2018). Material hardship was a categorical variable derived as an endorsement of yes to any of the 5‐items. Responses were dummy coded from yes/no to 1/0.

##### History of parental and family psychosocial adversity

History of parental anxiety/depression was assessed via syndrome scores from the Adult Self‐Report (ASR) subscale (Achenbach et al., 2017; Barch et al., 2018). History of prepandemic parental substance use was assessed from the ASR substance use subscale (see Appendix S1 Table S2). Parent report from the PhenX Family Environment Family Conflict Subscale assessed parental endorsement of conflict among family members on nine items (yes/no recoded to 1/0), and items were summed to obtain a total of items endorsed.

#### Measures during the COVID‐19 pandemic

##### Financial insecurity

Financial insecurity was assessed by two measures: (1) yes/no endorsement of loss of wages or a job due to the pandemic or (2) yes/no endorsement of material hardship experienced as a result of the pandemic using the same 5‐item material hardship questionnaire and scoring as the prepandemic timepoint. Responses were dummy coded to 1/0 for yes/no.

##### Social disruptions to routines

Four variables were used to index the extent of social disruption to routines: (1) parent help; (2) loss of contact with family/close friends; (3) disruptions to parent responsibilities; and (4) difficulty for youth completing school (see Appendix S1 Table S2). Parent help was a 3‐level categorical variable for (i) a single parent without help from a second caregiver, (ii) a supported parent who received regular help from another adult, or (iii) a supported parent experiencing loss of help from a second caregiver due to interference from the pandemic. Loss of contact with family and close nonfamily social contacts (friends, neighbors, members of a social, or religious group) was assessed via responses to a question with a 4‐point Likert scale ranging from “no change” to “severe,” assessing the degree by which parents reduced visits or contact with people as a result of social distancing. A measure of disruption to parent responsibilities was created by averaging parent responses to two items: whether caring for the child interfered with (i) work or (ii) household responsibilities (1: *none*, 2: *some*, and 3: *a great deal*). Difficulty completing school was assessed via parent response to a question inquiring on the difficulty for the child to complete remote schooling during the period of school closures on a 5‐point Likert scale ranging from “no problem” to “very hard.”

##### Adaptive coping

Parents reported adaptive coping activities during the pandemic, endorsing yes/no responses for exercise, making time to relax, hobbies, engaging in healthy behaviors like eating healthy and getting good sleep, taking breaks from television (TV) news or social media, taking care of the body, and connecting with others online or via phone. A composite comping score was created by summing endorsements across all coping activities (see Appendix S1 Figure S2 for correlations across items).

##### Parental alcohol use

Parents also reported on the frequency of alcohol use (i.e., days in the past month, ranging from 0 to 10 or more days). They were first asked how many days they consumed alcohol, ranging from 0 to 10 or more days. Endorsement of 1 or more days of alcohol use was proceeded by a follow‐up question assessing the number of drinks on a typical day when they consumed alcohol, ranging from 0 to 24 or more drinks. Parents were also asked how many days they had been drunk in the past month, ranging from 0 to 10 or more days.

### Statistical Analysis

Mixed‐effect linear regressions were used to test associations with each of the four pandemic family well‐being PCs. We included covariate fixed effects using a continuous variable for age and dummy coded categorical variables for sex (male: 1; female: 0), race (Black; Asian; or other/more than one race; reference level: White), ethnicity (1: Hispanic/Latinx; 0: non‐Hispanic/Latinx), study parent reporter (biological father; adoptive/custodial parent; another guardian; reference level: biological mother), and a random intercept of study site to control for between‐site correlations in the ABCD dataset. The sample analyzed did not contain siblings; therefore, a random effect for family was not included in the model. All continuous measures were centered to a mean of zero. Hierarchical linear regressions were implemented by creating a null model with the covariates only and then entering a block set of variables in succession as additional predictors, comparing each model to the previous model after each iteration using the log‐likelihood ratio test. In the first model comparison, we entered the prepandemic measures of psychosocial vulnerability as additional predictors of each of the four family well‐being PCs. In the next model, we entered all pandemic‐related measures of financial insecurity (loss of wages and material hardship) and social disruptions to routines as additional predictors of each of the family well‐being PCs, including all previous variables (i.e., covariates and prepandemic variables). Lastly, in the final model, the coping composite score and parental alcohol use were entered as additional predictors of each family well‐being principal component (PC), including all variables from the previous model (i.e., covariates, prepandemic variables, and pandemic financial insecurity and social disruption variables). Statistical models are described more in detail in Appendix S2. Missing data are reported in Appendix S1: Table S3 and S4, and a comparison between participants with and without missing data in the sample analyzed in Table S5, and a comparison of the sample analyzed with the full study sample in Table S6.

## RESULTS

### Family Well‐Being Principal Components

**Figure 1:**
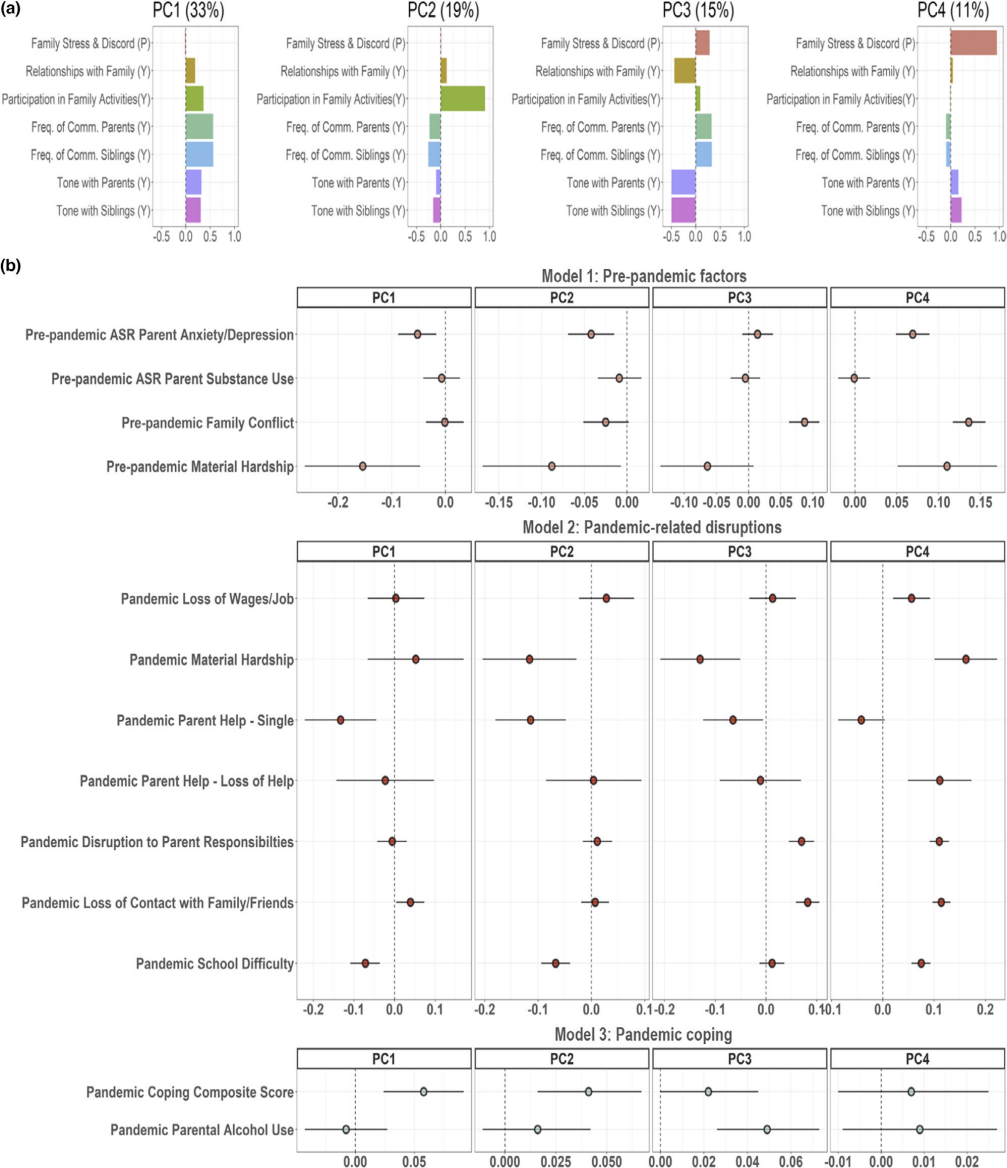
(a) PCA loadings for the four family well‐being principal components (PC). (b) Plots for standardized beta coefficients and 95% confidence intervals for each set of predictor variables entered into hierarchical mixed‐effect models, with model 1: all covariates and prepandemic factors; model 2: pandemic‐related financial and social disruptions, in addition to model 1 variables; and model 3: coping composite scores and parental alcohol use, in addition to all model 2 variables.

The top four PCs were selected as measures of family well‐being, statistically accounting for 78% of the variance across the seven variables entered into the PCA. The loadings for each PC are shown in Figure 1a. Plots for all PCs are provided in Appendix S1: Figure S3. The first PC loaded on youth report of higher frequency and a more positive tone of communication with family members and better family relationships, indicating youth‐perceived overall better family well‐being during the pandemic (33% variance explained). The second PC loaded on youth report of higher participation in family activities (19% variance explained). The third PC loaded on increased frequency of communication, relative to a worsening tone of communication, and worsening relationships, with higher scores indicating youth‐perceived worsening of family relationships (15% variance explained). The fourth PC loaded on parent report of increased family stress and discord (11% variance explained). Table 1 describes the distributions for the family well‐being PCs as well as all covariates and predictor variables.

**TABLE 1:** Description of Distribution of all Variables in Models for *N* = 4091 ABCD Participants Who Completed the COVID‐19 Parent Survey, With Prepandemic Data Acquired Between Baseline and the 2‐Year Follow‐up Visit (September 2018 to January 2020)

	Overall (*N* = 4091) *M* (*SD*) or *n* (%)
Covariates	
Youth Age (years)	12.57 (0.85)
Youth sex at birth	
Female	1936 (47.3)
Male	2155 (52.7)
Youth Race	
White	2866 (70.1)
Black	422 (10.3)
Asian	124 (3.0)
Other/Mixed	679 (16.6)
Youth ethnicity	
Hispanic	770 (18.8)
Parent reporter	
Biological Mother	3607 (88.1)
Biological Father	303 (7.4)
Adoptive/Custodial	147 (3.6)
Other	35 (0.9)
Prepandemic factors	
Parent ASR anxiety/Depression	4.94 (4.78)
Parent ASR substance use	1.21 (5.03)
Family conflict	2.37 (1.91)
Material hardship	491 (12.0)
Pandemic‐related factors	
Loss of Wages/Job	1978 (48.4)
Material hardship	495 (12.1)
Parent help	
Parent helped by a 2nd Caregiver	2903 (71.0)
Parent with loss of help from 2nd Caregiver	375 (9.2)
Single Parent	813 (19.9)
Loss of contact with family/friends	1.50 (0.72)
Difficulty with remote schooling	2.44 (1.12)
Disruption to parent responsibilities	1.67 (0.55)
Pandemic coping	
Coping composite score	4.25 (1.90)
Parental alcohol use days	2.51 (3.35)

ASR, Adult Self‐Report (Achenbach et al., 2017).

### Predictors of Pandemic Family Well‐Being PCs

The results of the hierarchical mixed‐effect model comparisons are shown in Table 2. For all PCs, additional variance was significantly and statistically attributable for each set of variables in model 1: prepandemic factors, model 2: pandemic‐related financial and social disruptions, and model 3: pandemic coping composite scores and parental alcohol use (see Table 2), suggesting a significant contribution from each additional set of factors, with the exception of model 3 (pandemic coping factors) for PC‐4. The individual standardized beta coefficients and 95% confidence intervals for each set of variables in each successive model are shown in Figure 1b. The standardized beta coefficients for the covariates are provided in Appendix S1: Figure S4.

**TABLE 2:** Results of Comparisons Using the Log‐Likelihood Ratio Test With Hierarchical Mixed‐Effect Linear Regressions for Each Set of Models Predicting the Four Principal Components (PCs) for Family Well‐Being During the Pandemic

Predictors	Overall Youth‐Perceived Family Well‐being (PC‐1)	Family Activities (PC‐2)	Youth‐Perceived Family Relationships (PC‐3)	Parent Perceived Family Stress & Discord (PC‐4)
Covariates only: age + sex + race + ethnicity + parent reporter	*R* ^2^ = .003	*R* ^2^ = .02	*R* ^2^ = .02	*R* ^2^ = .01
Model 1: covariates + prepandemic factors	*R* ^2^ = .007 χ^2^ (4, 4091) = 21.6 *p* < .001	*R* ^2^ = .03 χ^2^ (4, 4091) = 28.8 *p* < .001	*R* ^2^ = .04 χ^2^ (4, 4091) = 68.3 *p* < .001	*R* ^2^ = .08 χ^2^ (4, 4091) = 328.1 *p* < .001
Model 2: model 1 + pandemic financial & social/routine disruptions	*R* ^2^ = .01 χ^2^ (7, 4091) = 32.4 *p* < .001	*R* ^2^ = .04 χ^2^ (7, 4091) = 45.0 *p* < .001	*R* ^2^ = .06 χ^2^ (7, 4091) = 126.2 *p* < .001	*R* ^2^ = .08 χ^2^ (7, 4091) = 610.0 *p* < .001
Model 3: model 2 + pandemic coping	*R* ^2^ = .02 χ^2^ (2, 4091) = 10.9 *p* = .004	*R* ^2^ = .04 χ^2^ (2, 4091) = 10.5 *p* = .005	*R* ^2^ = .07 χ^2^ (2, 4091) = 22.6 *p* < .001	*R* ^2^ = .21 χ^2^ (2, 4091) = 1.2 *p* = .54

#### Youth Perception of Overall Family Well‐Being (PC‐1)

Higher overall youth‐perceived family well‐being (PC‐1) was higher among youth who identified as non‐Hispanic compared with Hispanic/Latinx youth (*p* < .001), with no other significant effects of age, sex, race, and parent reporter (all *p* > .08). Higher pandemic family well‐being PC‐1 scores (i.e., youth‐perceived overall better family well‐being) were predicted by less prepandemic psychosocial adversity, that is, no material hardship and lower parent ASR anxiety/depression scores (all β < −0.05, all *p* = .007), but not associated with prepandemic family conflict or parental substance use (*p* > .06). Youth‐perceived overall family well‐being (higher PC‐1 scores) was higher among youth experiencing less school difficulty and parents with help compared with single parents (all β < −0.05, all *p* ≤ .007). Increased loss of contact with family and friends was endorsed even among families reporting higher overall positive family well‐being (β < 0.04, *p* = .03). PC‐1 family well‐being scores were not associated with pandemic‐related material hardship, disruptions to parent responsibilities, or loss of wages/job, with no differences between parents who reported a loss of help from a second caregiver compared with a parent with help (all *p* > .38). Endorsement of a greater number of coping strategies was associated with better overall youth‐perceived family well‐being as indicated by higher PC‐1 scores (β = 0.06, *p* = .001), while there was no significant association for pandemic parental alcohol use (*p* = .68).

#### Family Activities (PC‐2)

There were sociodemographic differences in youth endorsement of family activities as indexed by PC‐2 scores such that younger youth, and youth who identified as Hispanic/Latinx (compared with non‐Hispanic youth) and youth who identified as Black or Other/Mixed (compared with youth identifying as White) showed lower family activity PC‐2 scores (*p* < .04), with adoptive caregivers reporting higher scores compared with biological mother reporters, and no other significant effects of sex, race, and parent reporter (all *p* > .18). Higher scores for family activities (PC‐2) were associated with lower prepandemic psychosocial adversity, that is, no material hardship and lower parent ASR anxiety/depression scores (all β < −0.04, all *p* < .03), but not with prepandemic family conflict or parental substance use (*p* > .06). Family activity PC‐2 scores were positively associated with lower difficulty completing remote schooling during the pandemic, with youth of single caregivers showing lower PC‐2 scores (i.e., less participation in family activities) compared with parents with regular help from second a caregiver (β < −0.11, all *p* = .001). Pandemic‐related material hardship was also associated with lower PC‐2 scores, suggesting youth with pandemic‐related material hardship endorsed lower participation in family activities (β = −0.10, *p* = .02). Family activity PC‐2 scores were not associated with disruptions to parent responsibilities, or loss of wages/job, with no differences between parents with regular help who reported a loss of help from a second caregiver compared with a parent with help (all *p* > .57). Endorsement of a greater number of coping strategies was associated with higher family activity PC‐2 scores (i.e., higher participation in family activities; β = 0.04, *p* = .003), with no significant association for pandemic parental alcohol use (*p* = .30).

#### Youth Perception of Worsening Family Relationships (PC‐3)

Higher PC‐3 pandemic family relationship scores (i.e., more severe worsening of family relationships) were endorsed by youth who identified as non‐Hispanic/Latinx (compared with Hispanic youth; *p* < .01) and among youth who identified as White (compared with youth who identified as Black or Other/Mixed; all *p* < .01), with biological mother reporters also showing higher PC‐3 scores compared with “other” caregivers (*p* = .02), and no other significant effects of age, sex, race, and parent reporter (all *p* > .07). Higher endorsement of prepandemic family conflict was associated with higher pandemic family relationship PC‐3 scores (β = 0.08, *p* < .001), suggesting prepandemic family conflict may be a risk factor for worsening of family relationships during the pandemic. Prepandemic parental anxiety/depression scores, parental substance use, or material hardship were not significantly associated with family well‐being PC‐3 scores (*p* > .08). Greater endorsement of loss of contact with family and friends and greater endorsement of disruption to parent responsibilities was associated with more severe worsening of family relationships as indicated by higher PC‐3 scores (all β > 0.07, all *p* < .001). There was no significant association between parent loss of help (compared with parents with no loss of help), or school difficulty and PC‐3 family relationship scores (all *p* > .30).

Interestingly, youth in households with single parents (compared with parents with help) and youth in households with an endorsement of pandemic‐related material hardship showed lower PC‐3 family relationship scores, indicating less severe worsening of family relationships as perceived by these youth. Furthermore, higher PC‐3 family relationship scores, that is, greater worsening of family relationships, was predicted by not only a greater endorsement of coping strategies (β = 0.02, *p* = .037) but also greater endorsement of parental alcohol use days (β = 0.05, *p* < .001). This indicated a greater parental engagement with all types of coping strategies was related to higher youth‐perceived worsening of family relationships and may reflect situations of greater stress for youth and families.

#### Parent Perception of Family Stress and Discord (PC‐4)

Higher PC‐4 scores (i.e., greater endorsement of parent‐perceived family stress and discord) were observed for older youth and youth who identified as White compared with youth who identified as Black or Asian (*p* < .02), with adoptive caregivers reporting lower PC‐4 scores (i.e., less family stress and discord) compared with biological mother reporters, and no other significant effects of age, sex, race, and parent reporter (all *p* > .18). Greater parent‐perceived family stress and discord, as measured by PC‐4 scores, was higher among parents with more prepandemic ASR anxiety/depression syndrome scores, greater endorsement of prepandemic family conflict, endorsement of prepandemic material hardship, and endorsement of pandemic‐related loss of wages (all β > 0.05, all *p* < .003), with no association with prepandemic parental substance use (*p* = .92). Greater PC‐4 family stress and discord scores were predicted by the endorsement of pandemic‐related material hardship, endorsement of greater pandemic‐related loss of contact with family and friends, greater parent disruption to routines, and greater difficulty completing remote schooling (all β > 0.03, all *p* < .001). There were no significant associations for the coping composite measure or parental alcohol use days with PC‐4 scores of family stress and discord scores (all *p* > .38).

## DISCUSSION

We studied family well‐being among 4091 demographically diverse youth and parents to understand the extent prepandemic psychosocial adversity and pandemic‐related factors of financial and social disruptions, including coping strategies, impacted family well‐being during the COVID‐19 pandemic. We used a PCA to derive four components describing unique aspects of family well‐being during the pandemic, including youth‐perceived overall family well‐being (PC‐1), family activity participation (PC‐2), youth‐perceived worsening of family relationships (PC‐3), and parent‐perceived family stress and discord (PC‐4). We found most components of family well‐being were negatively affected by prepandemic parent anxiety/depression and family conflict, as well as preexisting material hardship. Critically, most components of family well‐being were also negatively affected by pandemic‐related financial adversity, specifically, material hardship, and greater social disruptions, including parent difficulty completing work and home responsibilities, youth difficulty completing remote schooling, and increased loss of contact with family and friends.

Pandemic‐related material hardship was associated with lower youth‐perceived overall family well‐being, less family activities, and greater parent‐perceived family stress and discord. Pandemic‐related social disruptions such as interruptions to a parent's ability to complete home and work responsibilities and greater loss of contact with family and friends were related to worse family well‐being as indicated by elevated scores in youth‐perceived worsening of family relationships (PC‐3) and parent‐perceived family stress and discord (PC‐4). In contrast, increased difficulty completing school was associated with worse youth‐perceived overall family well‐being (PC‐1) and less participation in family activities (PC‐2), reflecting situations in which families may have experienced particularly taxing demands on resources. Lastly, greater engagement with coping activities during the pandemic was related to better overall youth‐perceived family well‐being and more family activities, while greater pandemic parental alcohol use was related with youth reported worsening of family relationships. Our findings point to important financial and social indicators affecting family well‐being during times of crises such as the COVID‐19 pandemic.

The first family well‐being component indicating overall youth perception of family well‐being reflected a pattern of interactions between family members (i.e., tone and frequency of communication, quality of relationships, and family activities) that according to the FAAR model may reflect exchanges in interactions that are an attempt to balance relationships as the family devotes resources to adjust to the crisis (Patterson, 2002). Youth who perceived worse overall family well‐being (PC‐1 scores) during the pandemic were in households with more prepandemic psychosocial adversity (i.e., material hardship and higher parental anxiety/depression), and had greater difficulty in completing remote schooling during closures, reflecting a greater difficulty in balancing family interactions. Overall family well‐being was more positive in households with a parent who had help from a second caregiver. Greater loss of contact with family and friends, that is, reduced visits or communication due to social distancing, was endorsed even among youth with overall better family well‐being, suggesting that the balancing of family interactions in this context was not contingent on social contact with others outside of their household.

In the context in which family interactions were more balanced in response to a crisis (PC‐1 scores), we found that more positive youth‐perceived family well‐being was related to a greater number of coping strategies (i.e., making time to relax, exercise, prioritizing sleep, and healthy nutrition). While we cannot determine a causal influence of these active coping strategies on more positive family well‐being, exercise and making time to relax may be active coping strategies that families can adopt to promote healthy well‐being through personal and family stress reduction (Grossman et al., 2004; Haglund et al., 2007). These findings in adolescents are consistent with previous work in adults suggesting coping activities like exercise and relaxation could help reduce distress during the COVID‐19 pandemic (Bateman et al., 2021; Brand et al., 2020; Conversano et al., 2020; Rodríguez‐Rey et al., 2020).

The second component of family well‐being measured endorsement of greater participation in family activities (PC‐2) for which scores were higher among youth with less prepandemic psychosocial adversity. Pandemic‐related material hardship was related to lower endorsement of family activities during the pandemic, consistent with past research suggesting that economic disadvantage for some families may result in fewer resources and less leisure time to dedicate to family activities (Orthner et al., 2004). Youth with lower family participation in activities were in households with overall fewer resources to cope with the challenges of the pandemic. Endorsement of family activities was highest among households with a parent who had help from a second caregiver compared with single caregivers. Households with single parents may have been impacted more by the challenges of the pandemic in terms of financial burden and childcare, contributing to a reduction in resources or time to dedicate to family activities. Similarly, households endorsing greater difficulty with youth remote schooling endorsed lower participation in family activities. In contrast, more participation in family activities was related to a greater endorsement in the number of coping strategies.

The third component of family well‐being was primarily youth perception of family relationships (i.e., higher frequency but worse tone and quality of communication) and reflected what is described by the FAAR model as disequilibrium and disorganization in the family. Families may reach this imbalance of interactions when families have maximized their resources in dealing with the demands of a crisis (Patterson, 2002). Consistent with this idea, youth who perceived more severe worsening of family relationships (PC‐3) were in households with less psychosocial resources, including a history of more family conflict before the start of the pandemic, greater endorsement of pandemic‐related disruptions to parent responsibilities due to caring for a child and greater endorsement of loss of contact with family and friends. However, youth among families with pandemic‐related material hardship and youth in single‐parent households reported less severe worsening of family relationships as indexed by lower PC‐3 scores. These findings are in line with the FAAR model, which posits that during times of crises, the experience of struggle can help all families emerge more resilient, regardless of potential risk for hardship. For instance, despite potential adversity, youth with material hardship reported better quality of relationships and tones of communication with family members compared with financially better off peers without material hardship. In addition, the FAAR model also suggests that for some individuals, the presence of some level of adversity allows individuals to acquire skills in coping and adapting strategies when faced with challenges. Future studies should investigate whether youth exposed to adversity during the pandemic (i.e., material hardship) but were also in environments with positive family well‐being, indeed do emerge stronger and better prepared for future challenges (Fergus & Zimmerman, 2005).

In terms of coping, we found greater parental alcohol use days, and to a lesser extent, a greater overall number of coping strategies were associated with youth‐perceived worsening of family relationships, including an increase in communication and negative tone. Previous studies have shown that adults under distress may be more vulnerable to adopt a maladaptive coping strategy such as alcohol or substance use (Veldhuis et al., 2021; Vetter et al., 2008; Vinkers et al., 2020). The associations for overall greater engagement of coping activities and parental alcohol use with higher youth‐perceived worsening of family relationships may reflect situations in which families may have maximized resources and indeed at disequilibrium in terms of family interactions in response to the challenges and distress brought on by COVID‐19 pandemic.

The final component of family well‐being encompassed parent‐perceived family stress and discord (PC‐4), for which scores were higher among families with prepandemic as well as pandemic‐related material hardship. More parent‐perceived family stress and discord was also predicted by a history of greater family conflict and parent anxiety/depression. This is consistent with studies on the financial impact on family well‐being during disasters, with our findings indicating material hardship (i.e., food and housing insecurity) were risk factors for greater family distress and discord (Gassman‐Pines et al., 2020; Prime et al., 2020; Seddighi et al., 2021; Williams & Cheadle, 2016). Parent family stress and discord was the only family well‐being component that was predicted by loss of wages during the pandemic, suggesting a narrower short‐term impact of lost wages, possibly due to financial support that families may have received from government stimulus payments (Casado et al., 2020). However, we did not specifically ask about the financial impact of the loss of wages or a job for families, higher‐earning families who reported a loss of wages may have been impacted less compared with lower‐earning families.

Parent‐perceived family stress and discord was predicted by almost all other pandemic‐related social disruptions, including more difficulty for youth to complete remote schooling, greater disruption to parent responsibilities as a result of caring for a child, greater loss of contact with family and friends, and also among parents who endorsed loss of help from a second caregiver. Parent family stress and discord more closely indexed parental response to the stressors and disruptions experienced as a result of the COVID pandemic, and our findings are limited in factoring the extent parents disclosed information or shared their perception of stress and discord with youth.

## STRENGTHS AND LIMITATIONS

In this study, we analyzed measures of family well‐being collected summer of 2020, during a time in which most families had experienced closures and disruptions to routines for several weeks, with most families likely impacted by COVID‐19‐related disruptions. The ABCD study subsample analyzed in this study differed slightly from that of the ABCD study baseline sample, with 2 percentage points less representation of youth who identified as Hispanic and 6 percentage points less representation of youth who identified as Black, indicating a lower response rate for families from an underrepresented race or ethnicity for the ABCD COVID‐19 survey. Importantly, in the sample analyzed, a comparison of participants with and without missing data showed the groups were mostly similar in demographics, with only slight differences in the representation of sex between groups (56% males in the missing data group compared with 51% males in the nonmissing data group). These relatively minimal differences in missing data were unlikely to influence the PCA‐estimated imputation of missing data. Although we observed small effect sizes by traditional standard, the range of effect sizes observed in this study are similar to the scale of effect sizes observed for large‐sample‐size studies like the ABCD Study, where 0.05 was the expected median effect size (Dick et al., 2021; Owens et al., 2021).

In addition, we only investigated a limited period in a prolonged crisis in a large cohort of diverse families. In our sample of parents (88% mothers), the degree to which caring for a child interfered with household and work responsibilities was strongly linked to poorer family well‐being. Our findings are consistent with reports that those caring for children, in particular women, have felt the most stressed during the pandemic (Cluver et al., 2020; Park et al., 2020). It is possible that family well‐being changed throughout the course of the pandemic, especially in response to changes in restrictions, return to work, and opening of schools, most adopting new normalcy with protocols to reduce the spread of infection (i.e., social distancing, continued masking, and hybrid school schedules), which altogether may have eased distress for some families while evoking continued or new distress for other families. Future studies can investigate changes in family well‐being throughout the course of the pandemic to understand factors that may promote positive family relationships and functioning, despite constant and wide social and financial changes. Some studies suggest the impact and experience of the pandemic among youth may differ by sociodemographic factors such as sex and race/ethnicity. For example, one study in the same cohort found that female youth showed higher levels of psychological distress than males (Kiss et al., 2022), and another found youth from underrepresented racial and ethnic groups experienced greater socio‐emotional adversity during the pandemic (Stinson et al., 2021). Given that underrepresented racial and ethnic groups have been disproportionately impacted by COVID‐19‐related disruptions (Shim & Starks, 2021), family well‐being may also differ among groups more affected by the pandemic. While direct investigation of differences by sex and race/ethnicity in family well‐being during the pandemic were beyond the scope of this study, these are important factors to be investigated in future analyses.

Another limitation is that we did not assess the intensity or frequency of engagement with coping activities which may also be related to family well‐being and vary for families with material hardship, such as flexibility in time to engage in leisure activities such as exercise (Cerin & Leslie, 2008). Similarly, while prepandemic substance use was not associated with any of the family well‐being outcome measures, it is possible that family well‐being may be sensitive to time‐varying changes in prepandemic parental substance use as well as the duration of the history of substance use (i.e., whether more recent use or more stable patterns of use differentially contribute to family dynamics). While parental alcohol use days were associated with one measure of family well‐being, parent‐perceived stress and discord, a limitation was the use of different measurements to assess parental substance use in the prepandemic and COVID protocol, making a direct comparison of pre‐ and postparental substance use difficult. Longitudinal investigations in changes in family well‐being concerning coping strategies, including adaptive and maladaptive, are needed to determine whether such coping strategies help families with severe financial insecurity emerge more resilient during the pandemic.

## CONCLUSION

During the COVID‐19 pandemic, many families experienced the compounded stress of financial hardship and social disruption to normal social routines. In our sample, families with preexisting psychosocial problems or material hardship were most at risk for worse family well‐being during the pandemic. Pandemic‐related factor of loss of access to family and friends, disruptions to parent responsibilities as a result of caring for children, and greater difficulty of completing remote schooling for youth during closures were associated with youth‐perceived worsening of family relationships and increased parent‐perceived stress and discord among family members. In turn, while pandemic‐related material hardship was associated with lower participation in activities and greater parent‐perceived family stress and discord, youth with material hardship also reported less severe perceived worsening of relationships. Overall, greater endorsement of coping strategies was associated with more positive family well‐being, while parental alcohol use was associated with youth‐perceived worsening of family relationships. These findings highlight the importance of mental health, financial support, and coping strategies to buffer COVID‐19‐related family distress for families with adolescents during and after the pandemic.

## ACKNOWLEDGEMENTS

Data used in the preparation of this article were obtained from the Adolescent Brain Cognitive Development^SM^ (ABCD) Study (https://abcdstudy.org), held in the NIMH Data Archive (NDA). This is a multisite, longitudinal study designed to recruit more than 10,000 children age 9–10 and follow them over 10 years into early adulthood. The ABCD Study® is supported by the National Institutes of Health and additional federal partners under award numbers U01DA041048, U01DA050989, U01DA051016, U01DA041022, U01DA051018, U01DA051037, U01DA050987, U01DA041174, U01DA041106, U01DA041117, U01DA041028, U01DA041134, U01DA050988, U01DA051039, U01DA041156, U01DA041025, U01DA041120, U01DA051038, U01DA041148, U01DA041093, U01DA041089, U24DA041123, and U24DA041147. A full list of supporters is available at https://abcdstudy.org/federal‐partners.html. Additional support for this work was made possible from supplements to U24DA041123 and U24DA041147, the National Science Foundation (NSF 2028680), and Children and Screens: Institute of Digital Media and Child Development Inc. A listing of participating sites and a complete listing of the study investigators can be found at https://abcdstudy.org/Consortium_Members.pdf. ABCD consortium investigators designed and implemented the study and/or provided data but did not necessarily participate in analysis or writing of this report. Dr. Gayathri Dowling was substantially involved in all of the cited NIH grants. This manuscript reflects the views of the authors and may not reflect the opinions, views, official policy, or position of the U.S. Department of Health and Human Services, NIH, or ABCD consortium investigators. The ABCD data repository grows and changes over time. The ABCD data used in this report came from the ABCD 3.0 data release (DOI: 10.15154/1519007) and the ABCD COVID‐19 Survey First Data Release (DOI: 10.15154/1520584). DOIs can be found at https://nda.nih.gov/study.html?id=901 and https://nda.nih.gov/study.html?&id=1041. Marybel R. Gonzalez was supported by NIAAA 3U01AA021692‐09S1.

## CONFLICT OF INTEREST

We have no conflicts of interest.

## Supporting information


**Appendix S1.** Supplementary tables and figures.
**Table S1**. Description of individual family well‐being measures used to derive latent factors using principal components analysis (PCA) from the June 2020 COVID Survey.
**Table S2.** Description of all pre‐pandemic and pandemic‐related predictor variables tested in association with pandemic family well‐being components.
**Table S3.** Description of missing ABCD study data for the *N* = 6153 parent COVID survey responses.
**Table S4.** Description of missing data for the family well‐being variables for which data was imputed using a principal components analysis with the svdImpute algorithm for the *N* = 4092 sample analyzed.
**Table S5.** Comparison of sample demographics for sub‐samples with and without missing data for the COVID youth report out of the *N* = 4092 analyzed.
**Table S6.** Comparison of sample demographics of COVID sample *N* = 4092 analyzed compared to the ABCD study baseline sample of 11,875.
**Figure S1.** Diagram showing the availability of data and overlap for ABCD study pre‐pandemic data and COVID survey data.
**Figure S2.** Correlation between pre‐pandemic measures of material hardship, household, income, and psychosocial factors (family conflict, parental anxiety/depression, parental substance use).
**Figure S3**. Plot of individual scores for all seven principal components (PCs) and the variance explained by each PC, estimated using the svdImpute algorithm using the pcaMethods package in R.
**Figure S4**. Plot of standardized beta coefficients and 95% confidence intervals for all fixed effect covariates (with random effect of site not shown), for the family well‐being principal components (PCs), comprising the null model for the hierarchical linear mixed‐effect model comparisons.
**Appendix S2**. Description of statistical models.

## References

[jora12776-bib-0001] Achenbach, T. M. , Ivanova, M. Y. , & Rescorla, L. A. (2017). Empirically based assessment and taxonomy of psychopathology for ages 1½–90+ years: Developmental, multi‐informant, and multicultural findings. Comprehensive Psychiatry, 79, 4–18. 10.1016/j.comppsych.2017.03.006 28356192

[jora12776-bib-0002] Aldwin, C. (2007). Stress, coping, and development: An integrative perspective. Guilford Press. https://books.google.com/books?hl=en&lr=&id=u‐_wag7hW3oC&oi=fnd&pg=PP2&dq=Stress,+coping,+and+development.&ots=KVvxCxTHJr&sig=0d_fbaCNc2PUUhVxSOks1YKH0I8

[jora12776-bib-0003] Barch, D. M. , Albaugh, M. D. , Avenevoli, S. , Chang, L. , Clark, D. B. , Glantz, M. D. , Hudziak, J. J. , Jernigan, T. L. , Tapert, S. F. , Yurgelun‐Todd, D. , Alia‐Klein, N. , Potter, A. S. , Paulus, M. P. , Prouty, D. , Zucker, R. A. , & Sher, K. J. (2018). Demographic, physical and mental health assessments in the adolescent brain and cognitive development study: Rationale and description. Developmental Cognitive Neuroscience, 32, 55–66. 10.1016/j.dcn.2017.10.010 29113758 PMC5934320

[jora12776-bib-0004] Bateman, L. B. , Schoenberger, Y.‐M. M. , Hansen, B. , Osborne, T. N. , Okoro, G. C. , Speights, K. M. , & Fouad, M. N. (2021). Confronting COVID‐19 in under‐resourced, African American neighborhoods: A qualitative study examining community member and stakeholders' perceptions. Ethnicity & Health, 26(1), 49–67. 10.1080/13557858.2021.1873250 33472411 PMC7875151

[jora12776-bib-0005] Baumeister, R. F. , & Leary, M. R. (1995). The need to belong: Desire for interpersonal attachments as a fundamental human motivation. Psychological Bulletin, 117(3), 497–529. 10.1037/0033-2909.117.3.497 7777651

[jora12776-bib-0006] Bell, D. N. F. , & Blanchflower, D. G. (2020). US and UKlabour markets before and during the Covid‐19 crash. National Institute Economic Review, 252, R52–R69. 10.1017/nie.2020.14

[jora12776-bib-0007] Brand, R. , Timme, S. , & Nosrat, S. (2020). When pandemic hits: Exercise frequency and subjective well‐being during COVID‐19 pandemic. Frontiers in Psychology, 11, 2391. 10.3389/fpsyg.2020.570567 PMC7541696 33071902

[jora12776-bib-0008] Casado, M. G. , Glennon, B. , Lane, J. , Mcquown, D. , Rich, D. , & Weinberg, B. A. (2020). The effect of fiscal stimulus: Evidence from COVID‐19 (No. w27576). National Bureau of Economic Research. https://www.nber.org/system/files/working_papers/w27576/w27576.pdf

[jora12776-bib-0009] U.S. Census Bureau . (2020). Household Pulse Survey . https://www.census.gov/programs‐surveys/household‐pulse‐survey/data.html

[jora12776-bib-0010] Cerin, E. , & Leslie, E. (2008). How socio‐economic status contributes to participation in leisure‐time physical activity. Social Science & Medicine, 66(12), 2596–2609. 10.1016/J.SOCSCIMED.2008.02.012 18359137

[jora12776-bib-0011] Cluver, L. , Lachman, J. M. , Sherr, L. , Wessels, I. , Krug, E. , Rakotomalala, S. , Blight, S. , Hillis, S. , Bachman, G. , Green, O. , Butchart, A. , Tomlinson, M. , Ward, C. L. , Doubt, J. , & McDonald, K. (2020). Parenting in a time of COVID‐19. The Lancet, 395(10231), e64. 10.1016/S0140-6736(20)30736-4 PMC7146667 32220657

[jora12776-bib-0012] Conversano, C. , Di Giuseppe, M. , Miccoli, M. , Ciacchini, R. , Gemignani, A. , & Orrù, G. (2020). Mindfulness, age and gender as protective factors against psychological distress during COVID‐19 pandemic. Frontiers in Psychology, 11, 1900. 10.3389/fpsyg.2020.01900 33013503 PMC7516078

[jora12776-bib-0013] Cox, M. J. , & Paley, B. (1997). Families as systems. Annual Review of Psychology, 48(1), 243–267. 10.1146/annurev.psych.48.1.243 9046561

[jora12776-bib-0014] Czeisler, M. É. , Lane, R. I. , Petrosky, E. , Wiley, J. F. , Christensen, A. , Njai, R. , Weaver, M. D. , Robbins, R. , Facer‐Childs, E. R. , Barger, L. K. , Czeisler, C. A. , Howard, M. E. , & Rajaratnam, S. M. W. (2020). Mental health, substance use, and suicidal ideation during the COVID‐19 pandemic — United States, June 24–30, 2020. Morbidity and Mortality Weekly Report, 69(32), 1049–1057. 10.15585/mmwr.mm6932a1 32790653 PMC7440121

[jora12776-bib-0015] Dick, A. S. , Lopez, D. A. , Watts, A. L. , Heeringa, S. , Reuter, C. , Bartsch, H. , Fan, C. C. , Kennedy, D. N. , Palmer, C. , Marshall, A. , Haist, F. , Hawes, S. , Nichols, T. E. , Barch, D. M. , Jernigan, T. L. , Garavan, H. , Grant, S. , Pariyadath, V. , Hoffman, E. , … Thompson, W. K. (2021). Meaningful associations in the adolescent brain cognitive development study. NeuroImage, 239, 118262. 10.1016/j.neuroimage.2021.118262 34147629 PMC8803401

[jora12776-bib-0016] Fergus, S. , & Zimmerman, M. A. (2005). Adolescent resilience: A framework for understanding healthy development in the face of risk. Annual Review of Public Health, 26(1), 399–419. 10.1146/annurev.publhealth.26.021304.144357 15760295

[jora12776-bib-0017] Folkman, S. , & Moskowitz, J. T. (2012). Stress, appraisal, and coping. In Encyclopedia of health and behavior. SAGE Publications, Inc.. 10.4135/9781412952576.n198

[jora12776-bib-0018] Gassman‐Pines, A. , Ananat, E. O. , & Fitz‐Henley, J. (2020). COVID‐19 and parent‐child psychological well‐being. Pediatrics, 146(4). 10.1542/peds.2020-007294 PMC7546085 32764151

[jora12776-bib-0019] Grossman, P. , Niemann, L. , Schmidt, S. , & Walach, H. (2004). Mindfulness‐based stress reduction and health benefits: A meta‐analysis. Journal of Psychosomatic Research, 57(1), 35–43. 10.1016/S0022-3999(03)00573-7 15256293

[jora12776-bib-0020] Haglund, M. E. M. , Nestadt, P. S. , Cooper, N. S. , Southwick, S. M. , & Charney, D. S. (2007). Psychobiological mechanisms of resilience: Relevance to prevention and treatment of stress‐related psychopathology. Development and Psychopathology, 19(3), 889–920. 10.1017/S0954579407000430 17705907

[jora12776-bib-0021] Kantamneni, N. (2020). The impact of the COVID‐19 pandemic on marginalized populations in the United States: A research agenda. Journal of Vocational Behavior, 119, 103439. 10.1016/j.jvb.2020.103439 32390658 PMC7205696

[jora12776-bib-0022] Kiss, O. , Alzueta, E. , Yuksel, D. , Pohl, K. M. , de Zambotti, M. , Műller‐Oehring, E. M. , Prouty, D. , Durley, I. , Pelham, W. E. , McCabe, C. J. , Gonzalez, M. R. , Brown, S. A. , Wade, N. E. , Marshall, A. T. , Sowell, E. R. , Breslin, F. J. , Lisdahl, K. M. , Dick, A. S. , Sheth, C. S. , … Baker, F. C. (2022). The Pandemic's toll on young adolescents: Prevention and intervention targets to preserve their mental health. The Journal of Adolescent Health, 70(3), 387–395. 10.1016/J.JADOHEALTH.2021.11.023 35090817 PMC8789404

[jora12776-bib-0023] Lebow, J. L. (2020). Family in the age of COVID‐19. Family Process, 59(2), 309–312. 10.1111/famp.12543 32412686 PMC7273068

[jora12776-bib-0024] Masten, A. S. , & Motti‐Stefanidi, F. (2020). Multisystem resilience for children and youth in disaster: Reflections in the context of COVID‐19. Adversity and Resilience Science, 1(2), 95–106. 10.1007/s42844-020-00010-w 32838305 PMC7314620

[jora12776-bib-0025] Masten, A. S. , & Narayan, A. J. (2012). Child development in the context of disaster, war, and terrorism: Pathways of risk and resilience. Annual Review of Psychology, 63, 227–257. 10.1146/annurev-psych-120710-100356 PMC5858878 21943168

[jora12776-bib-0026] Newland, L. A. (2015). Family well‐being, parenting, and child well‐being: Pathways to healthy adjustment. Clinical Psychologist, 19(1), 3–14. 10.1111/cp.12059

[jora12776-bib-0027] Orthner, D. K. , Jones‐Sanpei, H. , & Williamson, S. (2004). The resilience and strengths of low‐income families. Family Relations, 53(2), 159–167. 10.1111/J.0022-2445.2004.00006.X

[jora12776-bib-0028] Otu, A. , Charles, C. H. , & Yaya, S. (2020). Mental health and psychosocial well‐being during the COVID‐19 pandemic: The invisible elephant in the room. International Journal of Mental Health Systems, 14, 1–5. 10.1186/s13033-020-00371-w 32514302 PMC7257210

[jora12776-bib-0029] Owens, M. M. , Potter, A. , Hyatt, C. S. , Albaugh, M. , Thompson, W. K. , Jernigan, T. , Yuan, D. , Hahn, S. , Allgaier, N. , & Garavan, H. (2021). Recalibrating expectations about effect size: A multi‐method survey of effect sizes in the ABCD study. PLoS One, 16(9 September), e0257535. 10.1371/journal.pone.0257535 34555056 PMC8460025

[jora12776-bib-0030] Park, C. L. , Russell, B. S. , Fendrich, M. , Finkelstein‐Fox, L. , Hutchison, M. , & Becker, J. (2020). Americans' COVID‐19 stress, coping, and adherence to CDC guidelines. Journal of General Internal Medicine, 35(8), 2296–2303. 10.1007/s11606-020-05898-9 32472486 PMC7259430

[jora12776-bib-0031] Patterson, J. (1993). The role of family meanings in adaptation to chronic illness and disability. https://psycnet.apa.org/record/1993‐97224‐010

[jora12776-bib-0032] Patterson, J. M. (1988). Families experiencing stress. I. the family adjustment and adaptation response model. II. Applying the FAAR model to health‐related issues for intervention and reasearch. Family Systems Medicine, 6(2), 202–237. 10.1037/H0089739

[jora12776-bib-0033] Patterson, M. J. (2002). Integrating family resilience and family stress theory. Journal of Marriage and Family, 64(May), 349–360.

[jora12776-bib-0034] Prime, H. , Wade, M. , & Browne, D. T. (2020). Risk and resilience in family well‐being during the COVID‐19 pandemic. American Psychologist, 75(5), 631–643. 10.1037/amp0000660 32437181

[jora12776-bib-0035] Rodriguez, L. M. , Litt, D. M. , & Stewart, S. H. (2020). Drinking to cope with the pandemic: The unique associations of COVID‐19‐related perceived threat and psychological distress to drinking behaviors in American men and women. Addictive Behaviors, 110, 106532. 10.1016/j.addbeh.2020.106532 32652385 PMC7320671

[jora12776-bib-0036] Rodríguez‐Rey, R. , Garrido‐Hernansaiz, H. , & Collado, S. (2020). Psychological impact and associated factors during the initial stage of the coronavirus (COVID‐19) pandemic among the general population in Spain. Frontiers in Psychology, 1540. 10.3389/fpsyg.2020.01540 32655463 PMC7325630

[jora12776-bib-0037] Russell, B. S. , Hutchison, M. , Tambling, R. , Tomkunas, A. J. , & Horton, A. L. (2020). Initial challenges of caregiving during COVID‐19: Caregiver burden, mental health, and the parent‐child relationship. Child Psychiatry and Human Development, 51(5), 671–682. 10.1007/s10578-020-01037-x 32749568 PMC7398861

[jora12776-bib-0038] Sameer, A. S. , Khan, M. A. , Nissar, S. , & Banday, M. Z. (2020). Assessment of mental health and various coping strategies among general population living under imposed COVID‐lockdown across world: A cross‐sectional study. Ethics, Medicine and Public Health, 15, 100571. 10.1016/j.jemep.2020.100571 32838000 PMC7386294

[jora12776-bib-0039] Schmits, E. , & Glowacz, F. (2021). Changes in alcohol use during the COVID‐19 pandemic: Impact of the lockdown conditions and mental health factors. International Journal of Mental Health and Addiction, 1–12, 1147–1158. 10.1007/s11469-020-00432-8 PMC7781407 33424513

[jora12776-bib-0040] Seddighi, H. , Salmani, I. , Javadi, M. H. , & Seddighi, S. (2021). Child abuse in natural disasters and conflicts: A systematic review. Trauma, Violence, and Abuse, 22(1), 176–185. 10.1177/1524838019835973 30866745

[jora12776-bib-0041] Shim, R. S. , & Starks, S. M. (2021). COVID‐19, structural racism, and mental health inequities: Policy implications for an emerging Syndemic. 10.1176/Appi.Ps.202000725, 72(10), 1193–1198. 10.1176/APPI.PS.202000725 33622042

[jora12776-bib-0042] Sohrabi, C. , Alsafi, Z. , O'Neill, N. , Khan, M. , Kerwan, A. , Al‐Jabir, A. , Iosifidis, C. , & Agha, R. (2020). World Health Organization declares global emergency: A review of the 2019 novel coronavirus (COVID‐19). International Journal of Surgery, 76, 71–76. 10.1016/j.ijsu.2020.02.034 32112977 PMC7105032

[jora12776-bib-0043] Stacklies, W. , & Redestig, H. (2007). The pcaMethods Package. *R Package Doc*., 1–11. http://bioconductor.jp/packages/2.14/bioc/vignettes/pcaMethods/inst/doc/pcaMethods.pdf 10.1093/bioinformatics/btm069 17344241

[jora12776-bib-0044] Steinberg, L. , & Silk, J. S. (2002). Parenting adolescents. In M. H. Bornstein (Ed.), Handbook of parenting: Children and parenting (pp. 103–133). Lawrence Erlbaum Associates Publishers. https://psycnet.apa.org/record/2002‐02629‐004

[jora12776-bib-0045] Stinson, E. A. , Sullivan, R. M. , Peteet, B. J. , Tapert, S. F. , Baker, F. C. , Breslin, F. J. , Dick, A. S. , Gonzalez, M. R. , Guillaume, M. , Marshall, A. T. , McCabe, C. J. , Pelham, W. E. , Van Rinsveld, A. , Sheth, C. S. , Sowell, E. R. , Wade, N. E. , Wallace, A. L. , & Lisdahl, K. M. (2021). Longitudinal impact of childhood adversity on early adolescent mental health during the COVID‐19 pandemic in the ABCD study cohort: Does race or ethnicity moderate findings? Biological Psychiatry Global Open Science, 1(4), 324–335. 10.1016/j.bpsgos.2021.08.007 34608463 PMC8479935

[jora12776-bib-0046] Sun, J. , Harris, K. , & Vazire, S. (2020). Is well‐being associated with the quantity and quality of social interactions? Journal of Personality and Social Psychology, 119(6), 1478–1496. 10.1037/pspp0000272 31647273

[jora12776-bib-0047] Tramonti, F. (2021). COVID‐19, systems thinking and the ecology of disease: A focus on the family. Journal of Evaluation in Clinical Practice, 27, 1172–1174. 10.1111/jep.13559 33734528 PMC8250525

[jora12776-bib-0048] Troyanskaya, O. , Cantor, M. , Sherlock, G. , Brown, P. , Hastie, T. , Tibshirani, R. , Botstein, D. , & Altman, R. B. (2001). Missing value estimation methods for DNA microarrays. Bioinformatics, 17(6), 520–525. 10.1093/bioinformatics/17.6.520 11395428

[jora12776-bib-0049] Veldhuis, C. B. , Nesoff, E. D. , McKowen, A. L. W. , Rice, D. R. , Ghoneima, H. , Wootton, A. R. , Papautsky, E. L. , Arigo, D. , Goldberg, S. , & Anderson, J. C. (2021). Addressing the critical need for long‐term mental health data during the COVID‐19 pandemic: Changes in mental health from April to September 2020. Preventive Medicine, 146, 106465. 10.1016/j.ypmed.2021.106465 33647353 PMC8136863

[jora12776-bib-0050] Verger, N. B. , Urbanowicz, A. , Shankland, R. , & McAloney‐Kocaman, K. (2021). Coping in isolation: Predictors of individual and household risks and resilience against the COVID‐19 pandemic. Social Sciences & Humanities Open, 3(1), 100123. 10.1016/j.ssaho.2021.100123

[jora12776-bib-0051] Vetter, S. , Rossegger, A. , Rossler, W. , Bisson, J. I. , & Endrass, J. (2008). Exposure to the tsunami disaster, PTSD symptoms and increased substance use – An internet based survey of male and female residents of Switzerland. BMC Public Health, 8(1), 1–6. 10.1186/1471-2458-8-92 18366682 PMC2277388

[jora12776-bib-0052] Vinkers, C. H. , van Amelsvoort, T. , Bisson, J. I. , Branchi, I. , Cryan, J. F. , Domschke, K. , Howes, O. D. , Manchia, M. , Pinto, L. , de Quervain, D. , Schmidt, M. V. , & van der Wee, N. J. A. (2020). Stress resilience during the coronavirus pandemic. European Neuropsychopharmacology, 35, 12–16. 10.1016/j.euroneuro.2020.05.003 32446705 PMC7211573

[jora12776-bib-0053] Volk, A. A. , Brazil, K. J. , Franklin‐Luther, P. , Dane, A. V. , & Vaillancourt, T. (2021). The influence of demographics and personality on COVID‐19 coping in young adults. Personality and Individual Differences, 168, 110398. 10.1016/j.paid.2020.110398 32952250 PMC7492069

[jora12776-bib-0054] Walsh, F. (2015). Strengthening family resilience. Guilford Press. https://books.google.com/books?hl=en&lr=&id=RY1_CgAAQBAJ&oi=fnd&pg=PP1&dq=Strengthening+family+resilience.&ots=ZltwAUGBA6&sig=yw245vvYyxN6gFyi9hFp2ZmuOGE#v=onepage&q=StrengtheningFamily resilience.&f=false

[jora12776-bib-0055] Walsh, F. (2016). Applying a family resilience framework in training, practice, and research: Mastering the art of the possible. Family Process, 55(4), 616–632. 10.1111/famp.12260 27921306

[jora12776-bib-0056] Wardell, J. D. , Kempe, T. , Rapinda, K. K. , Single, A. , Bilevicius, E. , Frohlich, J. R. , Hendershot, C. S. , & Keough, M. T. (2020). Drinking to cope during COVID‐19 pandemic: The role of external and internal factors in coping motive pathways to alcohol use, solitary drinking, and alcohol problems. Alcoholism: Clinical and Experimental Research, 44(10), 2073–2083. 10.1111/acer.14425 32870516

[jora12776-bib-0057] Williams, D. T. , & Cheadle, J. E. (2016). Economic hardship, parents' depression, and relationship distress among couples with young children. Society and Mental Health, 6(2), 73–89. 10.1177/2156869315616258 27942421 PMC5144156

[jora12776-bib-0058] World Health Organization . (2020). *Mental Health and Psychosocial Considerations During COVID‐19 Outbreak*. *WHO/2019*‐*nCoV/MentalHealth/2020.1* . https://www.who.int/docs/default‐source/coronaviruse/mental‐health‐considerations.pdf

[jora12776-bib-0059] Zaki, J. , & Craig Williams, W. (2013). Interpersonal emotion regulation. Emotion, 13(5), 803–810. 10.1037/a0033839 24098929

