# Supplementary material for: Family Well‐Being During the COVID‐19 Pandemic: The Risks of Financial Insecurity and Coping
Source: J Res Adolesc. 2022 Jun 24:10.1111/jora.12776. Online ahead of print. doi: 10.1111/jora.12776 (PMC9349420; doi:10.1111/jora.12776)
Supplement: Supplementary file 1 — Appendix S1. Supplementary tables and figures. Table S1. Description of individual family well‐being measures used to derive latent factors using principal components analysis (PCA) from the June 2020 COVID Survey. Table S2. Description of all pre‐pandemic and pandemic‐related predictor variables tested in association with pandemic family well‐being components. Table S3. Description of missing ABCD study data for the N = 6153 parent COVID survey responses. Table S4. Description of missing data for the family well‐being variables for which data was imputed using a principal components analysis with the svdImpute algorithm for the N = 4092 sample analyzed. Table S5. Comparison of sample demographics for sub‐samples with and without missing data for the COVID youth report out of the N = 4092 analyzed. Table S6. Comparison of sample demographics of COVID sample N = 4092 analyzed compared to the ABCD study baseline sample of 11,875. Figure S1. Diagram showing the availability of data and overlap for ABCD study pre‐pandemic data and COVID survey data. Figure S2. Correlation between pre‐pandemic measures of material hardship, household, income, and psychosocial factors (family conflict, parental anxiety/depression, parental substance use). Figure S3. Plot of individual scores for all seven principal components (PCs) and the variance explained by each PC, estimated using the svdImpute algorithm using the pcaMethods package in R. Figure S4. Plot of standardized beta coefficients and 95% confidence intervals for all fixed effect covariates (with random effect of site not shown), for the family well‐being principal components (PCs), comprising the null model for the hierarchical linear mixed‐effect model comparisons. Appendix S2. Description of statistical models. [file JORA-9999-0-s001.docx]

| **Parent Report** | | | |
| --- | --- | --- | --- |
| Measure | Variable Name | Question | Response Scale |
| Family Stress and Discord | fam_discord_cv | Please rate how much the coronavirus pandemic has changed your family's life in each of the following ways:  Stress and discord in the family: | **0,None**: Family members are showing no or very little signs of irritation or being short-tempered with one another  **1,** **Mild**: Family members occasionally irritable or short-tempered with one another, may occasionally shout at one another  **2,** **Moderate**: Family members frequently irritable and short-tempered with one another; and/or children in the home are throwing things, knocking over furniture, or showing signs of moderate stress  **3,** **Severe**: Family members are constantly irritable and short-tempered with one another, shouting frequently; children and adults in the home throwing things at one another, and/or knocking over furniture, or showing signs of severe stress |
| **Youth Report** | | | |
| Measure | Variable Name | Question | Response Scale |
| Quality of family relationships | relationships_w_family_cv | In the last week, the relationships between members of your family are: | 1, A lot worse \| 2, A little worse \| 3, About the same \| 4, A little better \| 5, A lot better |
| Participating in family activities | fam_act_cv | I participated in family activities... | 0, Never \| 1, Rarely \| 2, Occasionally \| 3, Frequently \| 4, Very frequently |
| Frequency of communication with parents | demo_parents_cv | How has the frequency of your communication changed during this time with parents? | 1, Noticeably Decreased \| 2, Somewhat Decreased \| 3, Has Not Changed \| 4, Somewhat Increased \| 5, Noticeably Increased \| 6, Not applicable |
| Frequency of communication with siblings | demo_siblings_cv | How has the frequency of your communication changed during this time with parents? | 1, Noticeably Decreased \| 2, Somewhat Decreased \| 3, Has Not Changed \| 4, Somewhat Increased \| 5, Noticeably Increased \| 6, Not applicable |
| Tone of communication with parents | demo_tone_parents_cv | How has the tone of your communication changed during this time with parents? | 1, Noticeably more negative \| 2, Somewhat more negative \| 3, Has not changed \| 4, Somewhat more positive \| 5, Noticeably more positive \| 6, Not applicable |
| Tone of communication with siblings | demo_tone_siblings_cv | How has the tone of your communication changed during this time with siblings? | 1, Noticeably more negative \| 2, Somewhat more negative \| 3, Has not changed \| 4, Somewhat more positive \| 5, Noticeably more positive \| 6, Not applicable |

**Appendix S1**

**Table S1**. Description of individual family well-being measures used to derive latent factors using principal components analysis (PCA) from the June 2020 COVID Survey.

**Table S2.** Description of all pre-pandemic and pandemic-related predictor variables tested in association with pandemic family well-being components.

|  | **Parent Report** | | | | |
| --- | --- | --- | --- | --- | --- |
|  | Measure | Variable Name | Question | Response Scale |  |
| Model 1 (pre-pandemic only) | Pre-pandemic Material Hardship | demo_fam_exp1_p, or demo_fam_exp2_p_l, or, demo_fam_exp3_p_l, or, demo_fam_exp4_p_l, or, demo_fam_exp5_p_l | Needed food but couldn't afford to buy it or couldn't afford to go out to get it?  Were without telephone service because you could not afford it?  Didn't pay the full amount of the rent or mortgage because you could not afford it?  Were evicted from your home for not paying the rent or mortgage?  Had services turned off by the gas or electric company, or the oil company wouldn't deliver oil because payments were not made? | Yes (1); No (0) |  |
|  | Pre-pandemic ASR Parent Anxiety/Depression | asr_scr_anxdep_r | Aseba Anxious/Depressed ASR Syndrome Scale (raw score) | 0::36 |  |
|  | Pre-pandemic ASR Parent Substance Use | Raw mean:  asr_q124_p,  asr_q125_p,  asr_q126_p | In the past 6 months…  About how many times per day dud you use tobacco  On how many days were you drunk  On how many days did you use drugs for nonmedical purposes (including marijuana, cocaine, and other drugs, except alcohol and nicotine) | 0::85 |  |
|  | Pre-pandemic Family Conflict | fes_p_ss_fc | fam_enviro1_p + fam_enviro2r_p + fam_enviro3_p + fam_enviro4r_p + fam_enviro5_p + fam_enviro6_p + fam_enviro7r_p + fam_enviro8_p + fam_enviro9r_p  <https://nda.nih.gov/data_structure.html?short_name=fes02> | 0, False  1, True |  |
| Model 2  (model 1 + pandemic financial insecurity) | Pandemic Loss of Wages | fam_wage_loss_cv | Since January 2020, has anyone in your household lost wages, sales, or work due to the impact of coronavirus on employment, business, or the economy? | 0, No  1, Yes |  |
|  | Pandemic Material Hardship | fam_exp2_cv, or,  fam_exp3_cv, or,  fam_exp4_cv, or,  fam_exp5_cv, or, fam_exp6_cv, or, fam_exp7_cv | 1. Needed food but couldn't afford to buy it or couldn't afford to get out to get it?  2.Were without telephone service because you could not afford it?  3. Didn't pay the full amount of the rent or mortgage because you could not afford it?  4. Were evicted from your home for not paying the rent or mortgage?  5. Had services turned off by the gas or electric company, or the oil company wouldn't deliver oil because payments were not made? |  |  |
| Model 3 (model 2 + pandemic social disruptions) | Pandemic Caregiver Status | caregiver_cv  caregiver_help_cv | Is there another adult who usually helps with caregiving responsibilities?  IF Yes,  Has the coronavirus situation interfered with the extent to which this person is helping? | 0, No  1, Yes |  |
|  | Pandemic Loss of Access to Friends/Family | fam_supp_acc_cv | Access to family and close non-family social contacts (such as friends, neighbors, members of a social or religious group you belong to) | 0, No change  1, Mild: Continued visits with social distancing and/or regular phone calls and/or televideo or social media contacts  2, Moderate: Loss of in person and remote contact with a few people, but not all supports  3, Severe: Loss of in person and remote contact with all supports |  |
|  | Disruption to Parent Responsibilities | Average:  household_ability_cv  work_ability_cv | Is your role caring for your child conflicting with your household responsibilities? | 1, None  2, Some  3, A great deal  999, Don’t Know |  |
|  |  |  | Is your role caring for your child conflicting with your work responsibilities? | 1, None  2, Some  3, A great deal  999, Don’t Know |  |
|  | Pandemic School Difficulty | school_difficulty | How easy or difficult was it for your child to complete school work remotely? | 1, No problem  2, Just some minor problems 3, Each day was different  4, Hard  5, Very Hard  6, NA  999, Don’t Know |  |
| Model 4 (model 3 + pandemic coping activities) | Pandemic Parental Alcohol Use Days | su_p_alc_use_cv | On how many days did you use alcohol? | 0, 0 \| 1, 1 \| 2, 2 \| 3, 3 \| 4, 4 \| 5, 5 \| 6, 6 \| 7, 7 \| 8, 8 \| 9, 9 \| 10, 10+ |  |
|  | Breaks from TV News/Social Media | p_cope_cv__1 | In the past week, to cope, have you done any of the following? (check all that apply) | Yes(1); No (0) |  |
|  | Caring for Body | p_cope_cv__3 |  | Yes(1); No (0) |  |
|  | Exercised | p_cope_cv__4 |  | Yes(1); No (0) |  |
|  | Hobbies | p_cope_cv__5 |  | Yes(1); No (0) |  |
|  | Healthy Nutrition/Sleep | p_cope_cv__6 |  | Yes(1); No (0) |  |
|  | Made time to relax | p_cope_cv__7 |  | Yes(1); No (0) |  |
|  | Connected with Other (online/phone) | p_cope_cv__8 |  | Yes(1); No (0) |  |

**Table S3.** Description of missing ABCD study data for the N = 6,153 parent COVID survey responses. Pre-pandemic data was pulled from the first available timepoint across all visits between 2018 and January 2020 (i.e., baseline, year 1, and year 2 visits).

|  | N missing |
| --- | --- |
| **Pre-pandemic Data** |  |
| Pre-pandemic Household Income | 480 |
| Pre-pandemic Material Hardship | 71 |
| Pre-pandemic ASR Parent Anxiety/Depression | 0 |
| Pre-pandemic ASR Parent Substance Use | 284 |
| Pre-pandemic Family Conflict | 0 |
| **COVID Parent Report** |  |
| Family Stress and Discord | 1,401 |
| Pandemic Loss of Wages | 691 |
| Pandemic Material Hardship | 690 |
| Pandemic Caregiver Status | 557 |
| Pandemic Loss of Access to Friends/Family | 1,401 |
| Pandemic Disruption to Household Responsibilities | 1,881 |
| Pandemic Disruption to Work Responsibilities | 1,885 |
| Pandemic School Difficulty | 2001 |
| Pandemic Parental Alcohol Use Days | 1,402 |
| Breaks from TV News/Social Media | 0 |
| Caring for Body | 0 |
| Exercised | 0 |
| Hobbies | 0 |
| Healthy Nutrition/Sleep | 0 |
| Made time to relax | 0 |
| Connected with Other (online/phone) | 0 |
| Note: ASR- Adult Self Report | |

**Table S4.** Description of missing data for the family well-being variables for which data was imputed using a principal components analysis with the svdImpute algorithm for the N = 4,092 sample analyzed.

|  | N missing |
| --- | --- |
| COVID-19 Survey Parent Report |  |
| Family Stress & Discord | 0 |
| COVID-19 Survey Youth |  |
| Quality of family relationships | 864 |
| Participating in family activities | 850 |
| Frequency of communication with parents | 959 |
| Frequency of communication with siblings | 1,182 |
| Tone of communication with parents | 943 |
| Tone of communication with siblings | 1,164 |

**Table S5.** Comparison of sample demographics for sub-samples with and without missing data for the COVID youth report out of the N = 4,092 analyzed. Note: we applied a PCA with imputed values for missing data for the youth report family well-being measures (n=1,305).

|  | **Sub-Sample with Complete Data** | **Sub-Sample with Missing Youth Report of Family Well-Being** | ***p*-value** |  |
| --- | --- | --- | --- | --- |
| n | 2786 | 1305 |  |  |
| Youth Age (mean (SD)) | 12.57 (0.85) | 12.58 (0.85) | 0.735 |  |
| Youth Sex at Birth = M (%) | 1426 (51.2) | 729 (55.9) | 0.006 |  |
| Race (%) |  |  | 0.510 |  |
| White | 1944 (69.8) | 922 (70.7) |  |  |
| Black | 293 (10.5) | 129 (9.9) |  |  |
| Asian | 91 (3.3) | 33 (2.5) |  |  |
| Other/Mixed | 458 (16.4) | 221 (16.9) |  |  |
| Hispanic = Yes (%) | 525 (18.8) | 245 (18.8) | 0.996 |  |
| Parent Reporter (%) |  |  | 0.146 |  |
| [BioMother] | 2466 (88.5) | 1140 (87.4) |  |  |
| [Adoptive/Custodial] | 88 (3.2) | 59 (4.5) |  |  |
| [BioFather] | 210 (7.5) | 93 (7.1) |  |  |
| [Other] | 22 (0.8) | 13 (1.0) |  |  |

**Table S6.** Comparison of sample demographics of COVID sample N = 4,092 analyzed compared to the ABCD study baseline sample of 11,875.

|  | **ABCD COVID Sample Analyzed** | **ABCD Study Baseline Sample** | ***p*-value** |  |
| --- | --- | --- | --- | --- |
| N | 4,091 | 11,878 |  |  |
| Youth Sex at Birth = M (%) | 2155 (52.7) | 6196 (52.2) | 0.583 |  |
| Race (%) |  |  | < 0.001 |  |
| White | 2866 (70.0) | 7525 (64.3) |  |  |
| Black | 422 (10.3) | 1869 (16.0) |  |  |
| Asian | 124 (3.1) | 276 (2.4) |  |  |
| Other/Mixed | 679 (16.6) | 2037 (17.4) |  |  |
| Hispanic = Yes (%) | 770 (18.8) | 2411 (20.6) | 0.018 |  |
|  |  |  |  |  |
|  |  |  |  |  |
|  |  |  |  |  |
|  |  |  |  |  |
|  |  |  |  |  |
|  |  |  |  |  |


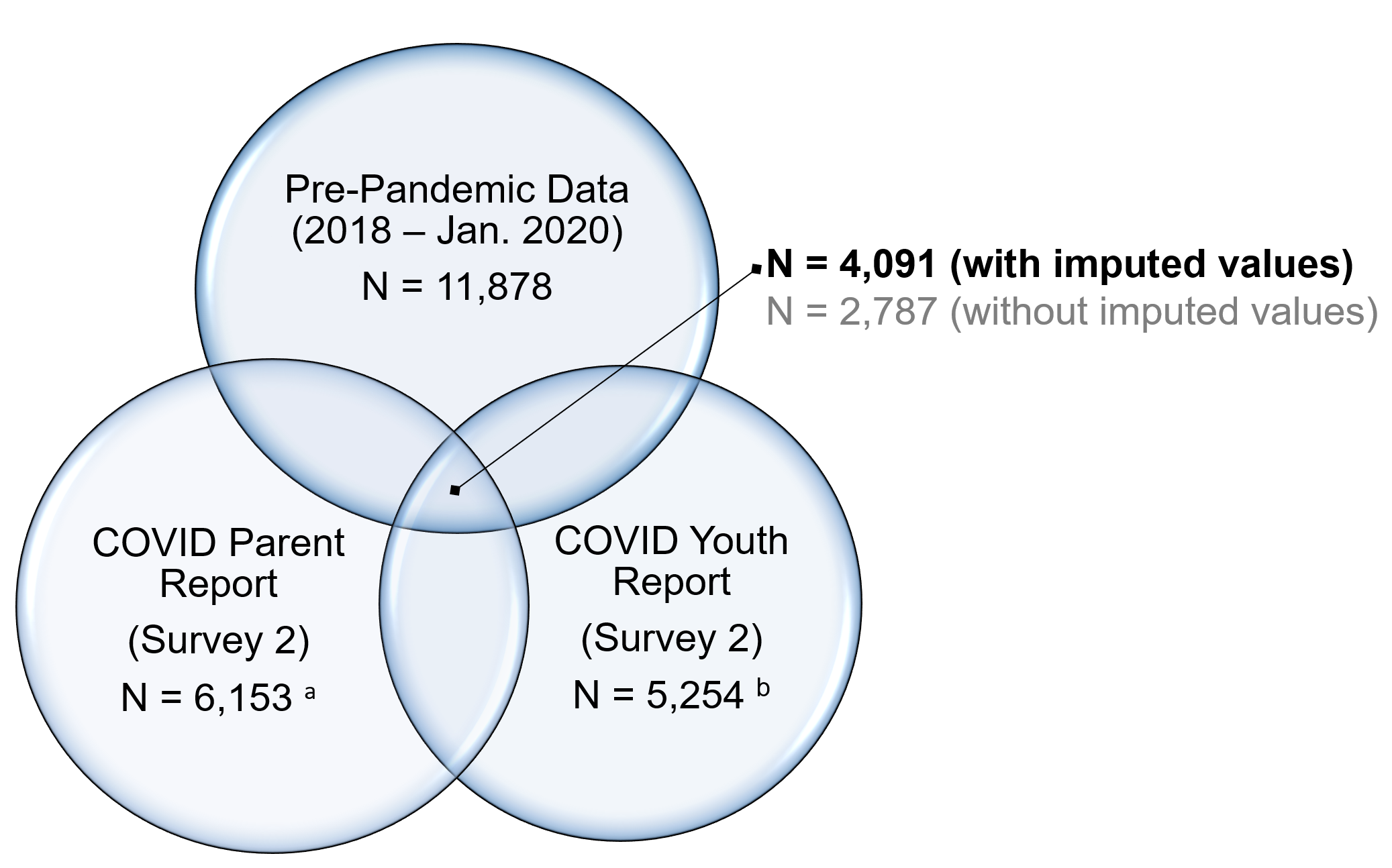


**Figure A1.** Diagram showing the availability of data and overlap for ABCD study pre-pandemic data and COVID survey data. Note: ^a^ see Table S3 for description of missing data for both pre-pandemic and pandemic variables for the N = 6,153 ABCD study participants for COVID parent report; ^b^ see Supplementary S4 for description of missing data imputed for youth COVID data.


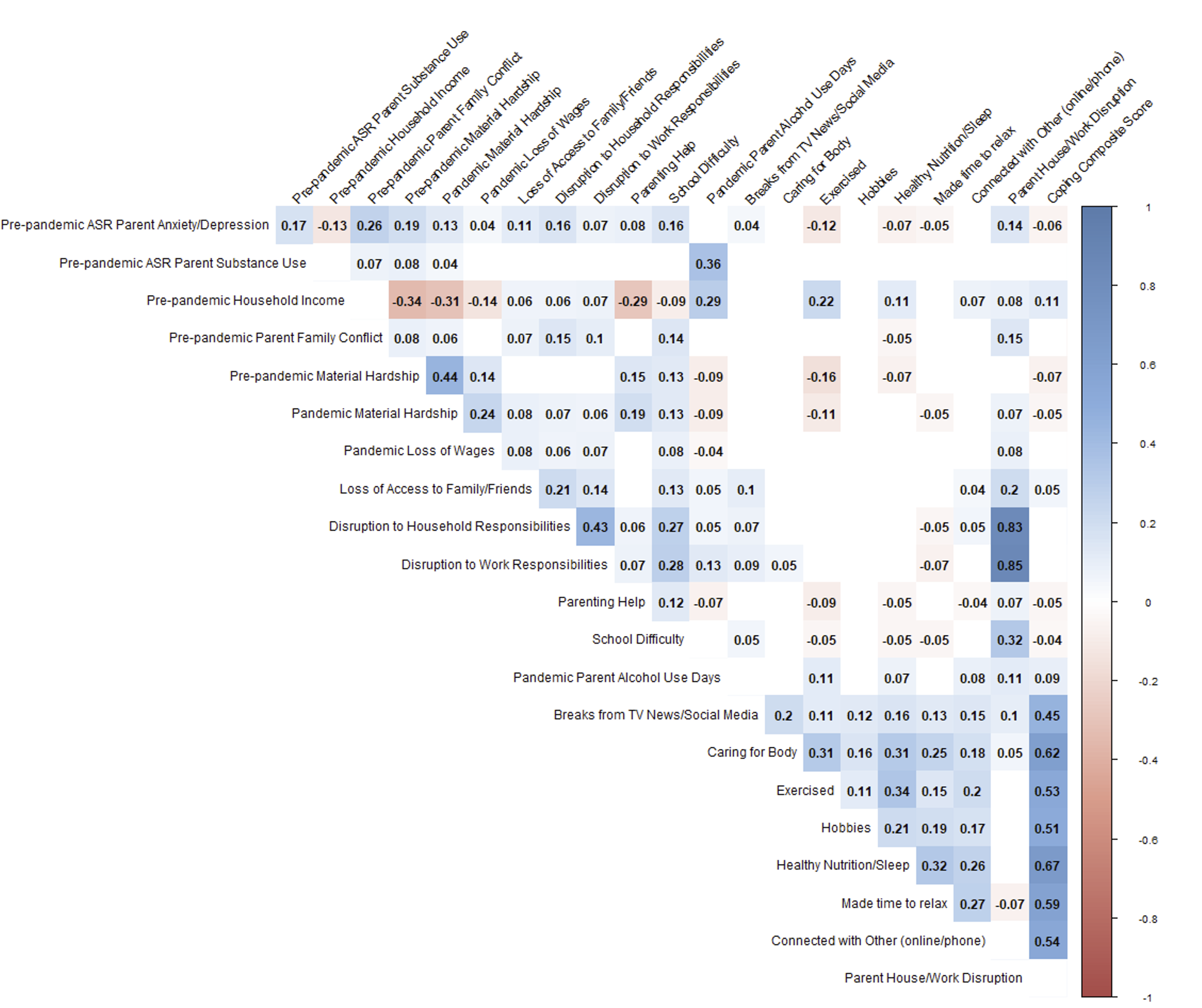


**Figure A2.** Correlation between pre-pandemic measures of material hardship, household, income, and psychosocial factors (family conflict, parental anxiety/depression, parental substance use). Note: pre-pandemic household income was not included in the main analyses due to high correlation with both pre and pandemic material hardship and also to reduce missing data (inclusion of household income would result in an additional N = 480 missing data points from sample analyzed).


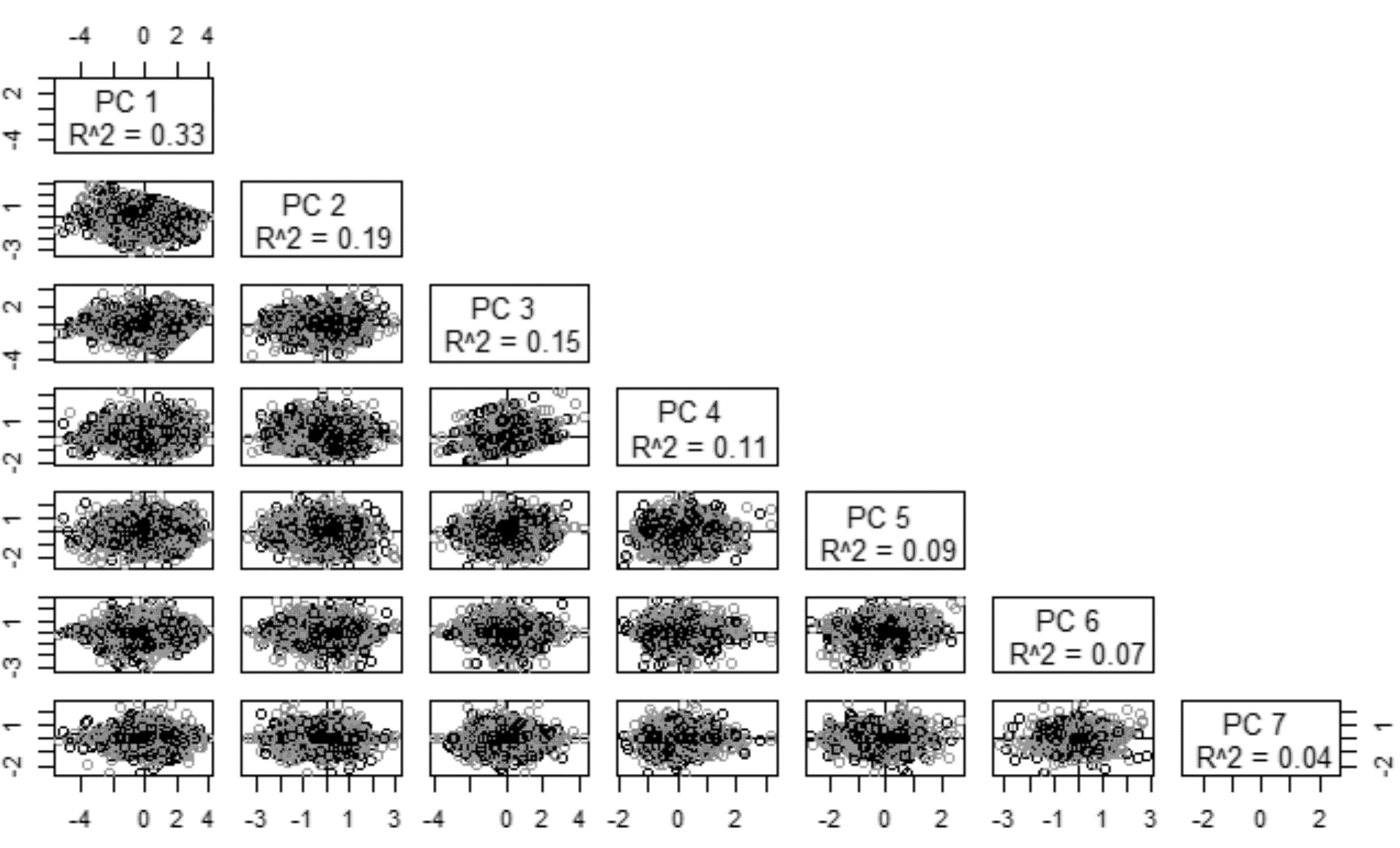


**Figure A3**. Plot of individual scores for all seven principal components (PCs) and the variance explained by each PC, estimated using the svdImpute algorithm using the pcaMethods package in R. Note: original PC 4 scores are shown here, and were then inverted (multiplied by -1) in the main analysis for ease of interpretation.


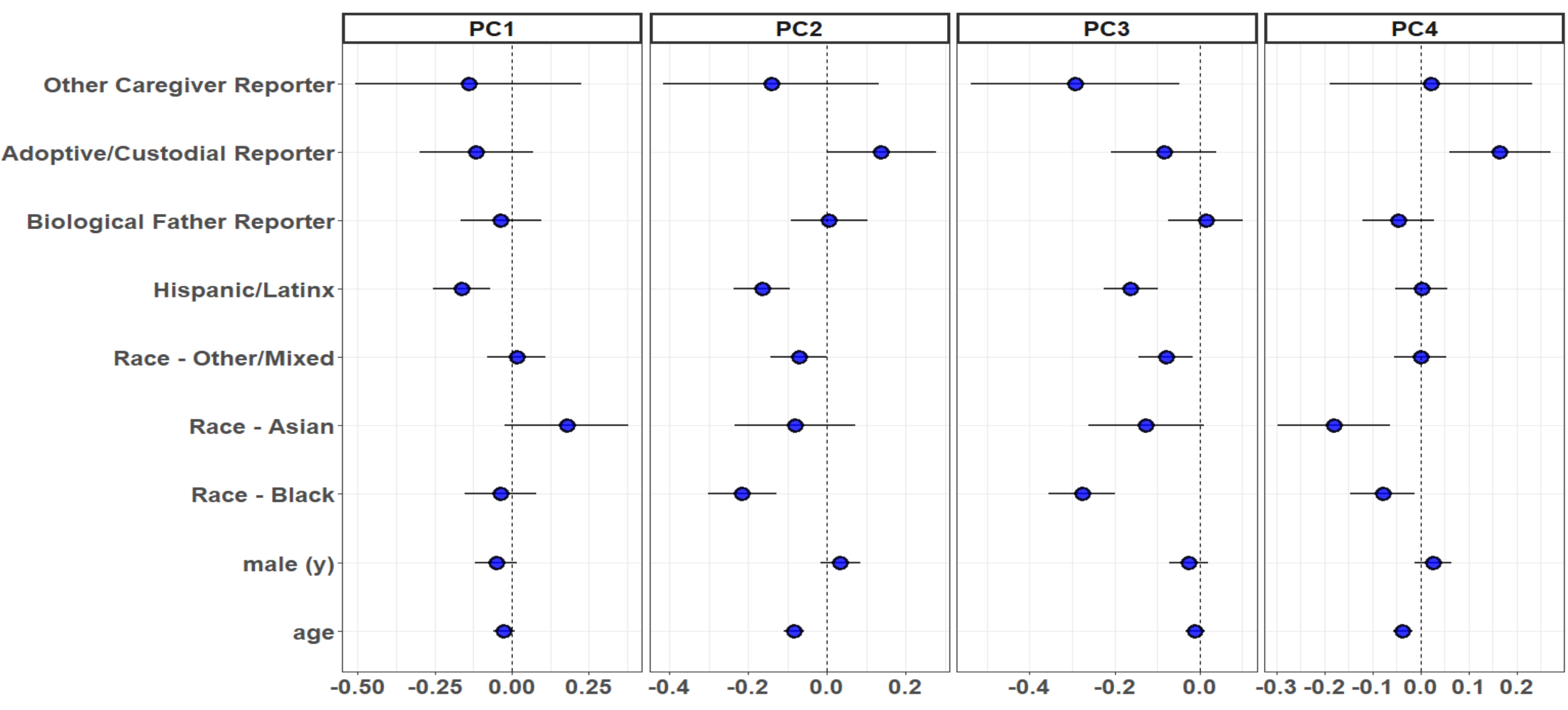


**Figure A4**. Plot of standardized beta coefficients and 95% confidence intervals for all fixed effect covariates (with random effect of site not shown), for the family well-being principal components (PCs), comprising the null model for the hierarchical linear mixed-effect model comparisons. Note: Reporter reference level is biological mother; Hispanic/Latinx reference level is Non-Hispanic/Latinx; Race reference level is White; and sex reference level is female.

**Appendix S2**

Description of Statistical Models

Model 1: The first model included each covariate variable (cov), and pre-pandemic independent variables (pre-pandemicIV), with the random intercept *u_i ,_* where *i* denotes the study site, for each *y* corresponding to each family well-being PC as a dependent variable:

y*i* ~ *β*_0_+*βX_i,_*_cov1_+...+*βX_i,_*_cov5_+*βX_i_*,_pre-pandemicIV1_+...+*βX_i_*,_pre-pandemicIV4_+*u_i_*+*e_i_*

Model 2: In the second model, in addition to the covariate variables (cov) and pre-pandemic independent variables (pre-pandemicIV), the pandemic independent variables (pandemicIV) were added as additional predictors, with the random intercept *u_i ,_* where *i* denotes the study site, for each *y* corresponding to each family well-being PC as a dependent variable:

y*_i_* ~ *β*_0_+*βX_i,_*_cov1_+...+*βX_i,_*_cov5_+*βX_i_*,_pre-pandemicIV1_+...+*βX_i_*,_prepandemicIV4_+*βX_i_*,_pandemicIV1_+...+*βX_i_*,_pandemicIV6_+*u_i_*+*e_i_*

Model 3: In the second model, in addition to the covariate variables (cov) and pre-pandemic independent variables (pre-pandemicIV), the pandemic independent variables (pandemicIV), the coping and the parental alcohol use variables were added as additional predictors, with the random intercept *u_i ,_* where *i* denotes the study site, for each *y* corresponding to each family well-being PC as a dependent variable:

y*_i_* ~ *β*_0_+*βX_i,_*_cov1_+...+*βX_i,_*_cov5_+*βX_i_*,_pre-pandemicIV1_+...+*βX_i_*,_prepandemicIV4_+*βX_i_*,_pandemicIV1_+... +*βX_i_*,_pandemicIV6_

+*βX_i_*,_coping IV_+*βX_i_*,_alcohol_ _use IV_+*u_i_*+*e_i_*
